# The Force–Frequency Characteristics of Quartz Wafers under a Cantilever Beam Structure

**DOI:** 10.3390/s24113359

**Published:** 2024-05-24

**Authors:** Junquan Shen, Chin-Yin Chen, Cheng-Yi Wu, Jiguang Cheng, Min-Chiang Chao, Qiang Zhou, Congda Lu

**Affiliations:** 1 College of Mechanical Engineering, Zhejiang University of Technology, Hangzhou 310023, China; sjq15167136560@163.com (J.S.); LCD@zjut.edu.cn (C.L.); 2 Zhejiang Key Laboratory of Robotics and Intelligent Manufacturing Equipment Technology, Ningbo Institute of Materials Technology and Engineer, Chinese Academy of Sciences, Ningbo 315201, China; 3 TXC (Ningbo) Corporation, Ningbo 315800, China; nchengyiwu@txc.com.tw (C.-Y.W.); njiguangcheng@txc.com.tw (J.C.); chaomk@txc.com.tw (M.-C.C.); nqiangzhou@txc.com.tw (Q.Z.)

**Keywords:** quartz wafers, force–frequency coefficient, cantilever beam

## Abstract

This study investigated the force–frequency characteristics of quartz wafers inside a cantilever beam frame. Firstly, the force–frequency coefficient formula of quartz wafers with fixed ends under axial force was analyzed. Firstly, the formula for the force–frequency coefficient of quartz wafers with fixed ends under axial force was analyzed. A force–frequency coefficient formula suitable for cantilever beam structures was derived by considering the changes in surface stress and stiffness of quartz wafers with fixed ends and one end under force on the other. Subsequently, the formula’s accuracy was verified by experiments, and the accuracy was more than 92%. In addition, strain simulation analysis was performed on three different shapes of quartz wafers, and experimental verification was carried out on two of them. The results revealed that trapezoidal quartz wafers and cantilever beam structures exhibited superior stress distribution to rectangular chips. Furthermore, by positioning electrodes at various locations on the surface of the quartz chip, it was observed that, as the electrodes moved closer to the fixed end, the force–frequency coefficient of the rectangular quartz chip increased, along with an increase in chip strain under the cantilever structure. In summary, this study provides a new approach for designing cantilever quartz resonator sensors in the future.

## 1. Introduction

With the rapid development of modern society, microforce values have been increasingly widely used in the analysis of mechanical properties in medicine, micro/nanomanufacturing, and microelectromechanical systems. In recent years, various microforce sensors have been designed and developed. For example, Liu et al. developed a tensile sensor based on a micro-springs structure [1], but its linearity could have been improved. In these domains, polyvinylidene fluoride (PVDF) sensors have been developed for cell injection applications, and atomic force microscopy (AFM) aids in measuring the mechanical properties of cells, organoids, and spheroids [2]. Shiro proposed a method for stiffness measurement using a quartz crystal resonator (QCR)-based force sensor probe [3]. Force feedback is required at each step in micro-assembly to ensure successful assembly [4]. In the micro-mechanical and nano-mechanical systems (MEMS/NEMS), measuring micro-forces is crucial for manipulating micro-objects. For instance, the primary components of optical communication network systems are microlenses, which micro-Newton (µN) level forces can easily damage. Currently, MEMS/NEMS also require detecting micro-contact forces during assembly to prevent damage caused by uncontrolled micro-forces [5]. Wu Rujing et al. proposed a micro-force sensor based on a fiber Bragg grating (FBG), which could be integrated into the ends of existing surgical instruments for direct force measurement [6]. The sensor demonstrated the potential to achieve millinewton-level measurement accuracy; however, it encountered challenges when addressing the measurement difficulty associated with the small instrument strain induced by axial forces.

In contrast, traditional piezoresistive or piezoelectric sensors commonly utilize silicon electronics microfabrication technology, restricting sensor materials to brittle substances like silicon and ceramics. While these sensors offer high testing accuracy, their testing range is constrained by the material’s intrinsic properties, such as the elastic limit. Conversely, quartz wafers exhibit rapid response times and can facilitate digital signal output with superior resolution and frequency. Additionally, they are less susceptible to signal noise and possess favorable static parameters, including linearity [7].

Quartz wafers, primarily composed of quartz (SiO_2_), typically form single crystals with distinct crystal structures and properties. They find extensive applications in various sectors, including electronics, communications, and computing. Quartz wafers are crucial components in oscillators and resonators within the electronics industry. Based on quartz’s stable physical and chemical characteristics, these wafers enable precise oscillation frequencies [8]. Therefore, they have been widely applied in clocks, timers, wireless communication devices, and computers internal clocks [9].

Quartz crystals have a prominent piezoelectric effect and are among the earliest discovered and most widely used piezoelectric crystals. Curie first discovered the piezoelectric effect of quartz crystals in 1880 and experimentally confirmed the inverse piezoelectric effect in 1881 [10,11]. The positive piezoelectric effect occurs when opposite charges appear on two opposing crystal surfaces, due to the mechanical deformation of crystals in a specific direction under external forces, known as polarization. Conversely, the inverse piezoelectric effect is observed when there is a proportional relationship between voltage and stress upon applying an external voltage at both ends of the crystal, resulting in mechanical stress. Essentially, the piezoelectric effect involves converting between electrical and mechanical energy, forming the theoretical basis for piezoelectric sensor development.

The piezoelectric equation delineates the correlation between parameters during the electromechanical conversion process of quartz crystals. Accounting for the anisotropy inherent in quartz crystals, the piezoelectric coefficient matrix and equation can characterize quartz crystals with diverse cuts. Quartz crystals undergo internal strain as elastomers when subjected to external forces, resulting in coupled mechanical and electrical responses during vibration. For clear-cut quartz wafers, the force–frequency characteristics will vary with the direction of the applied external forces [12]. The force–frequency coefficient is an essential parameter for measuring the performance of a mechanical sensor. Due to the rigidity of the structure connecting the chip and the external environment, the coefficient can be altered by designing the structure and selecting the material.

Our research has shown that, when secured at one end, quartz crystals form a cantilever beam structure as the sensor chip. Unlike crystals fixed at both ends, this configuration exhibits heightened sensitivity and enhanced compatibility for micro-force sensor advancement. These findings have significant implications for developing more efficient and sensitive sensor technologies.

By leveraging alterations in wafer strain and stiffness, this study adapted the current formula for determining the force–frequency coefficient of quartz wafers fixed at both ends to derive a formula applicable to cantilever quartz wafers. Experimental validation ensured the accuracy of the formula. Measurement of the force–frequency coefficient of a rectangular quartz wafer with adjustable electrode placement revealed enhanced force–frequency characteristics when the electrode was positioned to experience more significant strain. A simulation analysis was conducted on quartz wafers with different shapes in a cantilever beam structure. Research has found that triangles are more suitable for the cantilever structure of quartz wafers.

## 2. Materials and Methods

### 2.1. Force–Frequency Characteristics of Quartz under Force at Both Ends

**Figure 1:**
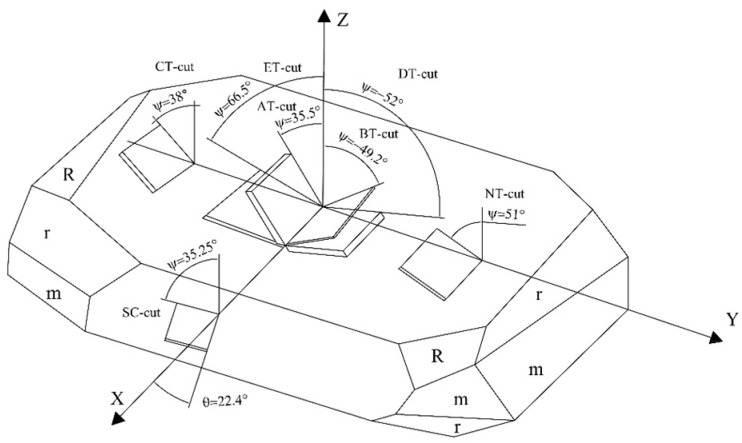
Quartz crystal cut.

Quartz, also known as crystals, is a vital piezoelectric material. When cut at different angles, the characteristics of quartz wafers vary depending on external factors. The quartz cut is shown in Figure 1. The AT cut, operating within the 0.5–350 MHz frequency range, represents a widely utilized thickness shear mode [13]. This cut displays minimal sensitivity to temperature at room temperature, resulting in a relatively slight frequency shift [14]. Moreover, it boasts straightforward processing, rendering it the predominant choice presently.

Conversely, the BT cut, another significant type of quartz thickness cut, displays heightened sensitivity to external temperature compared to AT-cut quartz wafers [15]. Furthermore, BT-cut wafers possess twice the thickness of AT-cut wafers at the same frequency, constraining their application range. Despite offering certain advantages, the SC cut poses challenges such as fragility, a restricted frequency range, a narrow application scope, a complex production process, and elevated costs [16]. Therefore, in this study, AT-cut quartz wafers were selected as the sensing unit to investigate the force–frequency characteristics of the cantilever structure.

Quartz exhibits remarkable force–frequency traits. In 1947, Bottom initially explored the force–frequency attributes of quartz wafers [17]. They noted that stress within the crystal could induce frequency disturbances and elucidated the correlation between force sensitivity and wafer cut. Following this, Ratajsk introduced the renowned Ratajsk coefficient to characterize the force-sensitive properties of resonators [18,19], as follows:

(1) $$K_{f}=\frac{\Delta f\cdot D\cdot n}{F_{S}\cdot f^{2}}$$

where $K_{f}$ represents the force sensitivity coefficient, which is related to the wafer’s cut and force direction, n is the order of harmonics, and D is the wafer diameter.

According to the principle that the resonance frequency Δf varies proportionally to the applied force $F_{s}$, the sensitivity coefficient $S_{S}$ before and after force application can be calculated as follows:

(2) $$S_{S}=\frac{\Delta f}{F_{S}}$$

The force–frequency coefficient formula for circular quartz wafers can be obtained, based on the formula above, as follows:

(3) $$S_{S}=\frac{K_{f}\cdot f^{2}\cdot \eta }{n\cdot D}$$

where η represents the correction coefficient for circular quartz wafers.

Fu Hao [20] compared the surface stress between square and circular quartz wafers with both ends fixed and revised the formula for calculating the axial force–frequency coefficient of square quartz wafers. His simulation findings unveiled a correlation between the surface stress of the two wafers when the diameter of the circular quartz wafer matched the short edge of the square quartz wafer. Through thorough analysis, Fu Hao observed that the surface stress ratio eventually stabilized, leading to the derivation of the formula describing the relationship between the surface stress ratio and the length–width ratio of the square quartz wafer as follows:

(4) $$y=1.95-0.6\sqrt{\frac{W}{L}}$$

where y denotes the ratio of surface stress between the square and circular quartz wafers, W represents the width of the quartz crystal, and L represents the length of the quartz crystal.

Fu’s study is significant because it addresses the axial direction issue in computing the force–frequency coefficient of square quartz wafers, thereby furnishing a dependable theoretical foundation for related domains. The revised formula, meticulously compared for accuracy and applicability with experimental data, instills confidence in its precision and reliability. Subsequently, the formula for determining the force–frequency coefficient of circular quartz wafers was adapted to derive the formula for calculating the force–frequency coefficient of square quartz wafers. This formula can be expressed as follows [21]:

(5) $$S_{1}=\frac{K_{f}\cdot f^{2}}{W\cdot n}\cdot {\left(1.95-0.6\sqrt{\frac{W}{L}}\right)}^{-1}$$

Equation (5) applies to square quartz wafers fixed at both ends and under axial loading. However, this paper mainly aims to calculate square quartz wafers’ force–frequency coefficients in a cantilever beam structure. Due to the piezoelectric effect of quartz, the strain of quartz wafers will affect their force–frequency characteristics. When computing the force–frequency coefficient, it is imperative to account for the impact of quartz strain on the output frequency of quartz wafers. Additionally, the boundary conditions of quartz wafers influence the force–frequency characteristics of quartz wafers. Upon investigating the effects of boundary conditions on the force–frequency coefficient of quartz, Zhou [21] discovered the following relationship between the stiffness of the package structure, the force–frequency coefficient of the package structure, the stiffness of the bare quartz wafer, and the force–frequency coefficient as follows [21]:

(6) $$\delta_{r}\cdot S_{r}=\delta_{m}\cdot S_{m}$$

where $\delta_{r}$ represents the stiffness of the quartz wafer, $S_{r}$ represents the force–frequency coefficient of the quartz wafer, $\delta_{m}$ represents the stiffness of the package structure, and $S_{m}$ represents the force–frequency coefficient of the package structure.

Two conditions are necessary for Equation (6) to be valid. Firstly, the maximum variation in the package structure under forces should approximately equal the maximum variation in the quartz wafer within the package structure. Secondly, suppose condition $\delta_{m}<\delta_{S}$ is met. In that case, the force–frequency coefficient of the package structure will exceed that of the crystal, which contradicts the frequency output of the quartz crystal, as the experimentally obtained force–frequency coefficient is based on the crystal’s coefficient. Therefore, Equation (6) can only be applied when the stiffness meets condition $\delta_{m}\geq \delta_{S}$, meaning that the stiffness of the package structure should surpass that of the crystal.

The force–frequency coefficient calculation Equation (5) is applied to wafers fixed at both ends. The correction primarily involves comparing the surface stress of square and circular wafers to adapt the formula for square quartz wafers. However, this correction solely addresses the quartz wafer with fixed ends, overlooking the variation in quartz strain between scenarios with fixed ends and those with a cantilever beam structure. Due to the difficulty in applying the calculation method of Equation (5) to quartz wafers with cantilever beam structures, it is necessary to introduce strain as a correction parameter to obtain a modified force–frequency coefficient calculating formula, suitable for quartz wafers with cantilever beam structures.

### 2.2. Force–Frequency Characteristics of Quartz in Cantilever Beam Structure

As the structure shifts from being fixed at both ends to a cantilever beam arrangement, the change in force application causes a decline in the stiffness of the quartz wafer. According to Equation (6), with the diminishing stiffness of the quartz crystal structure, there is an escalation in the force–frequency coefficient. Therefore, when modifying Equation (5), it is necessary to consider the influence of the stiffness to ensure that the revised force–frequency coefficient calculation equation is accurate.

The study provides significant insights into the force–frequency characteristics of quartz wafers with a cantilever beam structure. Introducing the strain correction parameter presents a more comprehensive and applicable method for calculating the force–frequency coefficient. Furthermore, a detailed analysis of the impact of reduced stiffness of quartz wafer structures on the force–frequency coefficient can enhance understanding and elucidate the response mechanism of cantilever quartz wafers under force loading. This offers valuable references and guidance for future research and applications. For a cantilever quartz wafer with one end fixed and the other end free, the force–frequency coefficient can be expressed as follows:

(7) $$S_{2}=\frac{K_{f}\cdot f^{2}}{W\cdot n}\cdot {\left(1.95-0.6\sqrt{\frac{W}{L}}\right)}^{-1}\cdot K_{1}\cdot K_{2}$$

where $K_{1}$ denotes the strain correction parameter and $K_{2}$ denotes the stiffness correction coefficient. We investigate a cantilever beam with one end fixed and the other free to move, aiming to ascertain the strain in the x-direction when the beam experiences a free force $F_{1}$ [22].

**Figure 2:**
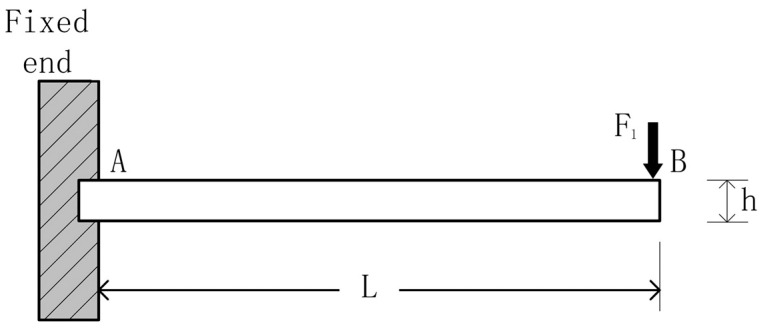
Cantilever beam subjected to a concentrated force at the free end.

As shown in Figure 2, M is the bending moment at a distance *x* from the fixed end *A*, as follows [23]:

(8) $$M\left(x\right)=F_{1}\left(L-x\right)$$

When the width $b\left(x\right)$ of the cantilever beam varies along its length, the moment of inertia $I\left(x\right)$ at a distance of x from the fixed end is determined as follows:

(9) $$I\left(x\right)=\frac{b\left(x\right)h^{3}}{12}$$

where F is the loading force, x is the distance from point A to be solved along the beam direction to the required point, L denotes the total length of the cantilever beam, and b and h are the width and height of the beam, respectively. Assuming that the strain along the cantilever beam width remains constant and the deflection is minimal, it is evident that, when x=L, the bending moment at the free end of the cantilever beam is zero. Typically, the tensile stress experienced by the beam can be expressed as follows [23]:

(10) $$\sigma =\frac{M\left(x\right)}{I\left(x\right)}c=Ec\frac{\partial^{2}u}{\partial x^{2}}$$

where c denotes the distance from the beam’s neutral axis to the point being analyzed (for a cantilever beam with constant height, c remains consistent along the x direction), $\frac{\partial^{2}u}{\partial x^{2}}$ represents the second derivative of the beam deflection, and E signifies the elastic modulus of the beam material. Equation (11) illustrates the relationship between the bending strain ε at any position x and beam curvature R, as follows:

(11) $$\varepsilon =\frac{c}{R}$$

The elastic modulus of the material is as follows:

(12) $$E=\frac{\sigma }{\varepsilon }$$

So, the axial strain on the neutral axis is as follows:

(13) $$\varepsilon_{1}\left(x\right)=\frac{M\left(x\right)}{IE}c$$

In this context, the second derivative of the cantilever beam equals the reciprocal of the curvature radius, i.e., $\frac{\partial^{2}u}{\partial x^{2}}=\frac{1}{R}$, which means that, in the cantilever beam structure, the axial strain $\varepsilon_{1}$ can ultimately be expressed as follows:

(14) $$\varepsilon_{1}=\frac{6F_{1}\left(L-x\right)}{Ebh^{2}}$$

For a beam with fixed ends and under axial pressure, the beam stress $\varepsilon_{2}$ is the strain when the beam is subjected to axial forces at both ends. $\varepsilon_{2}$ can be expressed as follows:

(15) $$\varepsilon_{2}=\frac{\Delta L}{L}$$

where L represents the length of the beam and ΔL represents the change in length when subjected to the force $F_{2}$.

According to Hooke’s law, within the elastic limit of the body, the stress and the strain become proportional, and the proportional coefficient is called Young’s modulus E. E can be expressed as follows:

(16) $$E=\frac{F_{2}L}{S\Delta L}$$

where S=bh represents the area under the influence of force $F_{2}$. The axial strain $\varepsilon_{2}$ can be expressed as follows:

(17) $$\varepsilon_{2}=\frac{F_{2}}{Ebh}$$

The strain correction coefficient $K_{1}$ can be expressed by the ratio of the strain $\varepsilon_{1}$ of the cantilever beam structure to the strain $\varepsilon_{2}$ of the wafer with both ends fixed, as follows:

(18) $$K_{1}=\frac{\varepsilon_{1}}{\varepsilon_{2}}=6\frac{L-x}{h}$$

The stiffness correction coefficient $K_{2}$ is primarily influenced by the quartz stiffness, which correlates with the length of the quartz wafer. Therefore, $K_{2}$ is predominantly associated with the quartz length. Experimental trials were performed on a quartz wafer with a fundamental frequency of 25 MHz, a length of 3.5 mm, and a width of 1.8 mm, to assess its force–frequency coefficient. Analyzing the variations in the force–frequency coefficient, adjustments were made to the formula for calculating the force–frequency coefficient of quartz.

**Figure 3:**
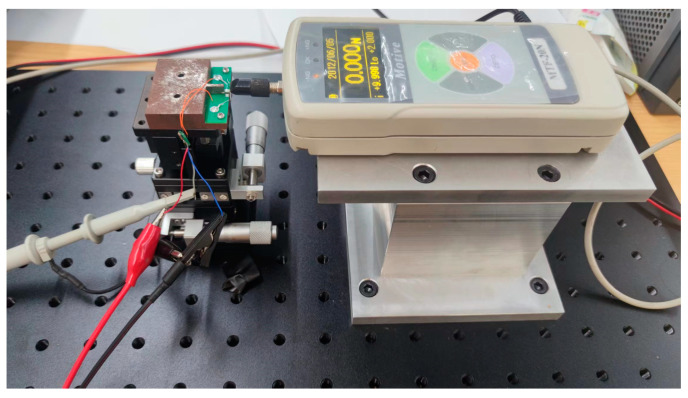
Experimental device for force measurement (MTF-2N).

**Figure 4:**
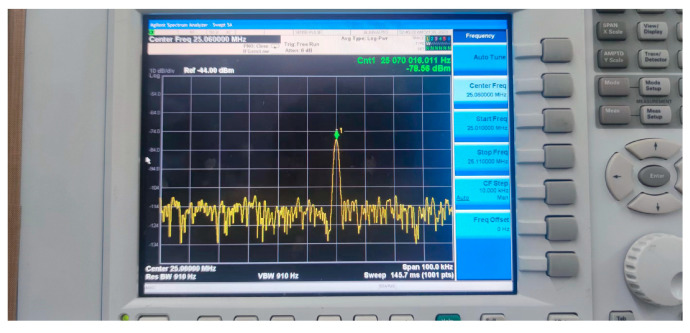
Spectrum analyzer (N9020A-503).

The quartz crystal wafers used for testing are AT-cut quartz wafers. The quartz wafers come in two shapes: rectangular and trapezoidal. The dimensions of the rectangular wafers are 3.5 mm × 1.8 mm, 6.5 mm × 2 mm, and 25.5 mm × 10 mm, with a fundamental frequency of 25 MHz. The trapezoidal quartz wafers have a top base length of 5 mm, a bottom base length of 15 mm, and a height of 25.5 mm, with a fundamental frequency of 8 MHz. We set up the experiment on an optical breadboard, installing a thrust meter (MTF-2N) and an XYZ axis displacement stage (LGD40) to measure the force applied to the sample. By moving the position of the displacement stage, the force exerted on the quartz crystal mounted on it was varied. Simultaneously, the quartz crystal’s real-time frequency changes were recorded using a spectrum analyzer (N9020A-503, Agilent Technologies Inc., Santa Clara, CA, USA) connected through an oscillation circuit, allowing the calculation of the force–frequency coefficient. The experimental force-measuring device is shown in Figure 3 and Figure 4.

**Figure 5:**
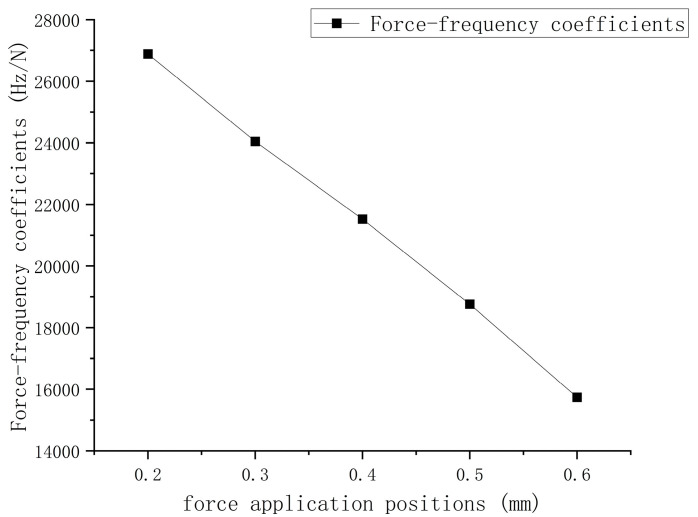
Force–frequency coefficients of quartz under different force application positions.

In practice, it is difficult to apply a concentrated force to the free end of the cantilever beam, as shown in Figure 2, so the force–frequency coefficient at x=L can be inferred by using a concentrated force to the quartz wafer at different positions.

(19) $$x_{1}=L-x$$

where $x_{1}$ is the distance between the force application position and the free end. The force–frequency coefficients of the quartz wafer at $x_{1}=$ 0.2 mm, 0.3 mm, 0.4 mm, 0.5 mm, and 0.6 mm were tested, and the experimental results are shown in Figure 5. The dots and line represent the measured force–frequency coefficients and the fitted line at different force application positions. Figure 5 shows that the force–frequency coefficients varied with the change in the force application position $x_{1}$ and had a linear relationship with $x_{1}$, as follows:

(20) $$S_{2}=-27582x_{1}+32424$$

A linear correlation was observed between the distance from the force position to the free end and the force–frequency coefficient; a more significant distance corresponded to a smaller force–frequency coefficient. When $x_{1}=0$, the force was applied at the free end, and the force–frequency coefficient of the quartz wafer was 32,424 Hz/N.

By substituting the measured force–frequency coefficient of the quartz wafer into Equation (7), the stiffness correction coefficient $K_{2}$ can be expressed as follows:

(21) $$K_{2}=\frac{L}{39W}$$

where *L* and *W* are the length and width of the quartz wafer, respectively.

By applying the formula for calculating the force–frequency coefficient of quartz wafers fixed at both ends, we can derive the formula for quartz wafers with one fixed end and one free end as follows:

(22) $$S_{2}=\frac{K_{f}\cdot f^{2}}{W\cdot n}\cdot {\left(1.95-0.6\sqrt{\frac{W}{L}}\right)}^{-1}\cdot 6\frac{L-x}{h}\cdot \frac{L}{39W}$$

A quartz wafer with a length of 6.5 mm, a width of 2 mm, and a fundamental frequency of 25 MHz was used to verify the modified force–frequency coefficient formula by varying the fixed-end length. First, the method in Figure 5 is used to determine the force–frequency coefficients at $x_{1}=0$ for different cantilever beam structures. The force–frequency coefficients for different sizes were obtained from the experimental results.

### 2.3. The Shape of Quartz Wafers

The above research revised the formula for calculating the force–frequency coefficient of cantilever quartz wafers. While testing the force–frequency coefficient of square wafers, it was observed that the force application position had a notable influence on the measured force–frequency coefficient range. Consequently, based on the properties of quartz, we devised a strategy to stabilize its force–frequency coefficient.

Quartz crystals demonstrate the piezoelectric effect, where deformation under external force leads to internal polarization, resulting in positive and negative charges on opposite surfaces of the material. As the external force fluctuates, the surface charge varies, termed the positive piezoelectric effect. Conversely, the inverse piezoelectric effect describes the strain generation in quartz crystals when subjected to an electric field and the subsequent elimination of strain when the electric field is removed. Piezoelectric sensors are developed based on this phenomenon. At present, square quartz wafers are primarily used in cantilever structures, but the strain distribution in square quartz wafers under cantilever structures could be more uniform. Due to the piezoelectric effect, quartz wafer deformation can affect the electric field output. Therefore, to maximize the output, the utilization rate of the strain should be maximized, and the measured force–frequency coefficient range should be stabilized. This section discusses the strain distribution of quartz wafers of other shapes under a cantilever beam structure.

**Figure 6:**
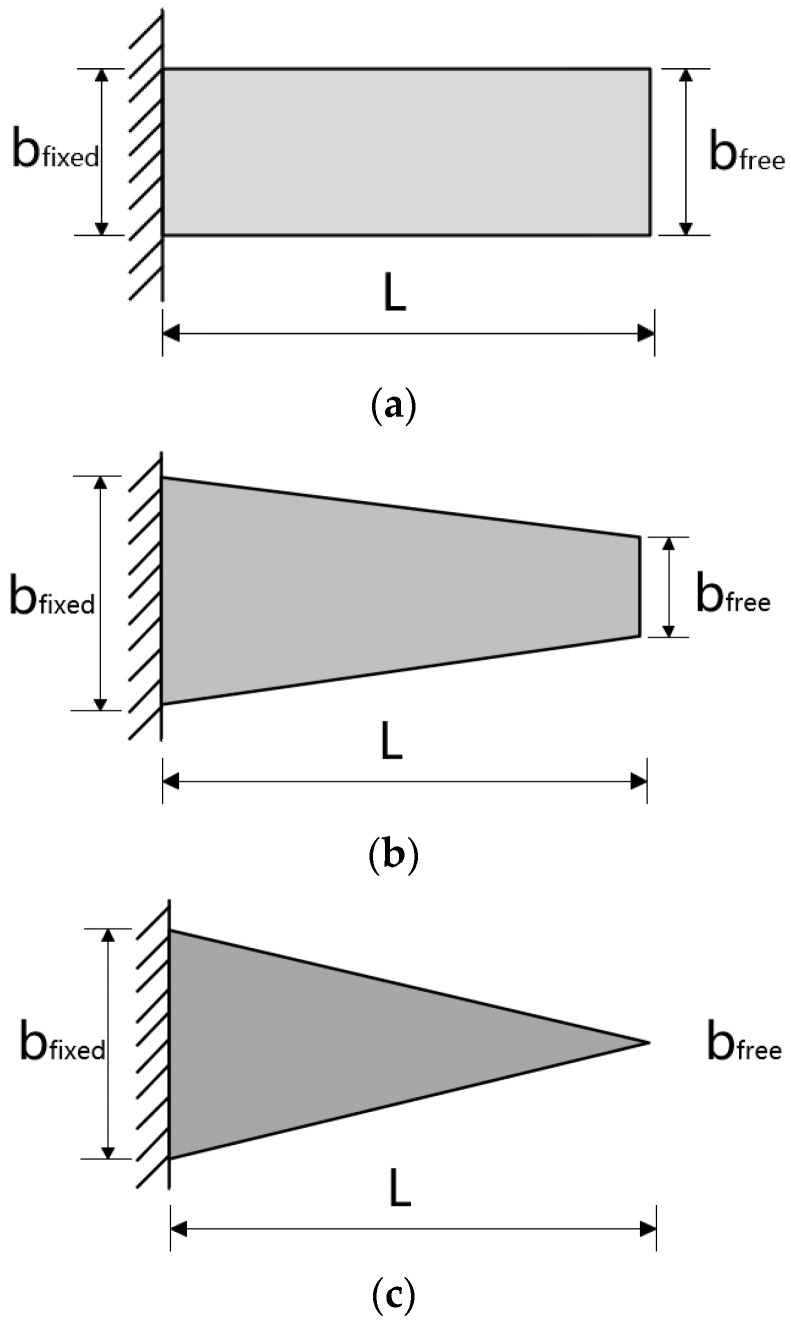
Size design of quartz wafers under different cantilever beam structures. (**a**) Rectangular structure. (**b**) Trapezoidal structure. (**c**) Triangular structure.

The strain variations in the material were examined when the free ends of rectangular, trapezoidal, and triangular quartz wafers were subjected to a concentrated force within a cantilever beam structure. In these three structures, the length L of the quartz wafers was fixed at 25.5 mm, and the height h was consistent at 0.2 mm. The specific dimensions of the three quartz wafers are shown in Figure 6 and Table 1 below.

ANSYS Workbench 2021 simulation software was subsequently used to simulate the three structures of the quartz wafers. Quartz was the chosen material, with a density, Young’s modulus, and Poisson’s ratio of 2650 g/cm^3^, 72 GPa, and 0.31, respectively. Fixed constraints were applied to the fixed end of each quartz wafer, with an equivalent concentrated force exerted on the free end.

The width of quartz wafers varied among the different structures, leading to distinct strain distributions. Moreover, due to differences in beam size and profile, direct strain comparison between the beams could have been more feasible. Hence, normalization was essential, utilizing the maximum strain on the structure as the reference and dividing the strain at each point along the centerline by this reference. Notably, a red area was evident at the fixed end of each structure, indicating the highest stress. The relative strain after normalization is expressed as follows [23]:

(23) $$\varepsilon_{n}(x)=\frac{\varepsilon (x)}{\varepsilon_{m}}$$

**Figure 7:**
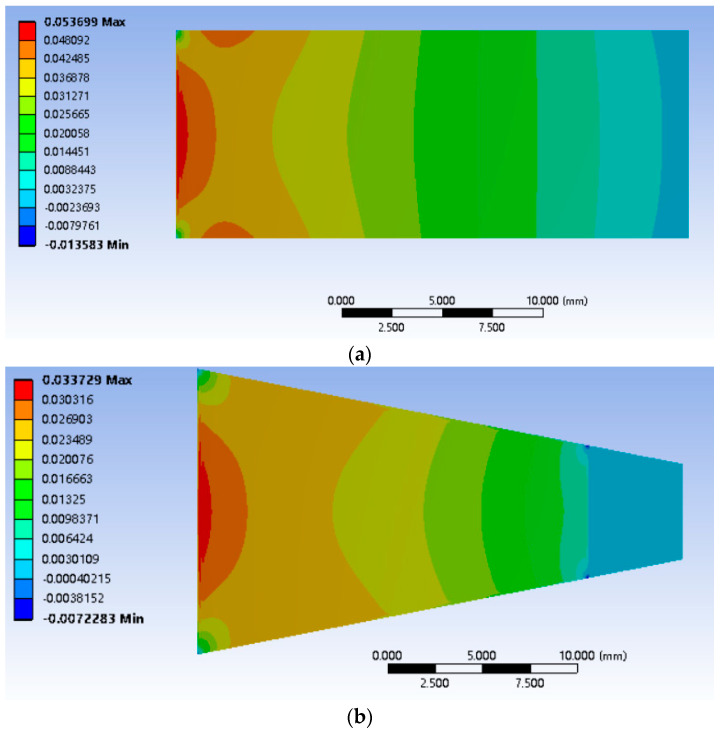

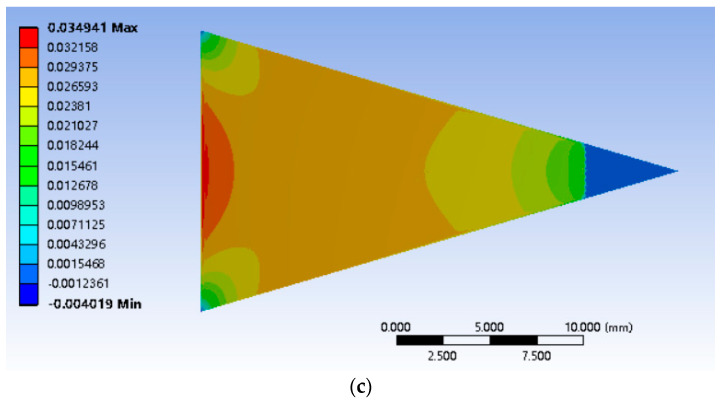
Stress simulation results of different shapes of quartz wafers. (**a**) Rectangular structure. (**b**) Trapezoidal structure. (**c**) Triangular structure.

**Figure 8:**
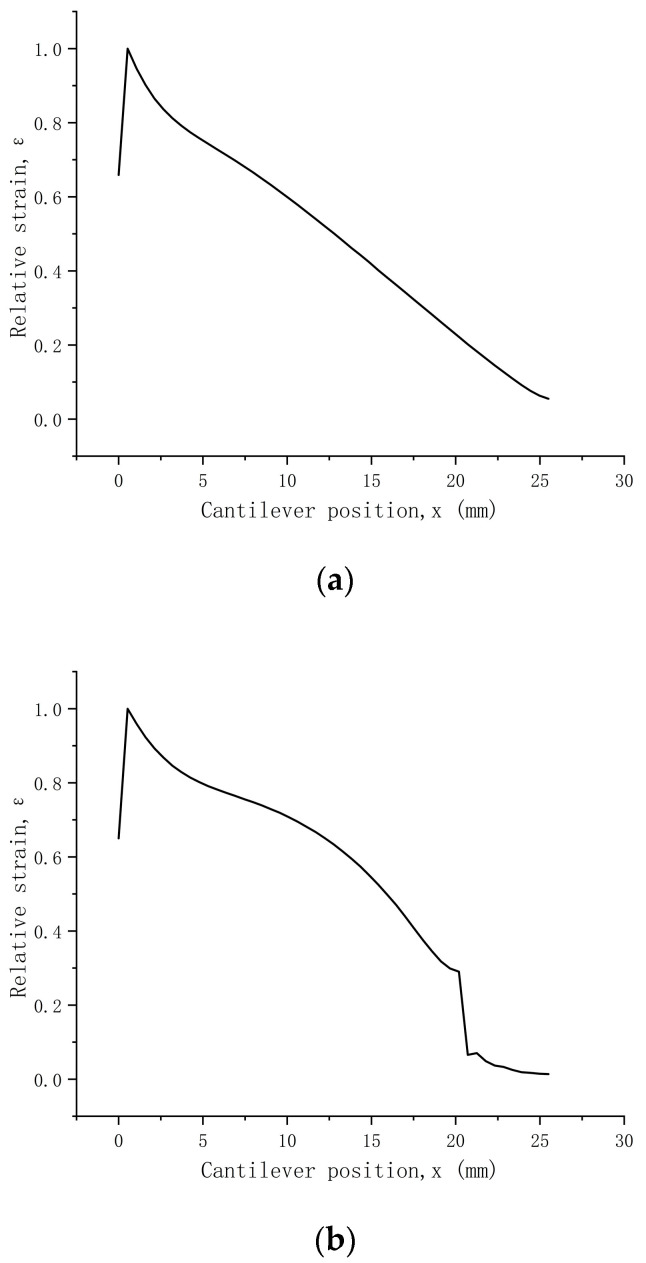

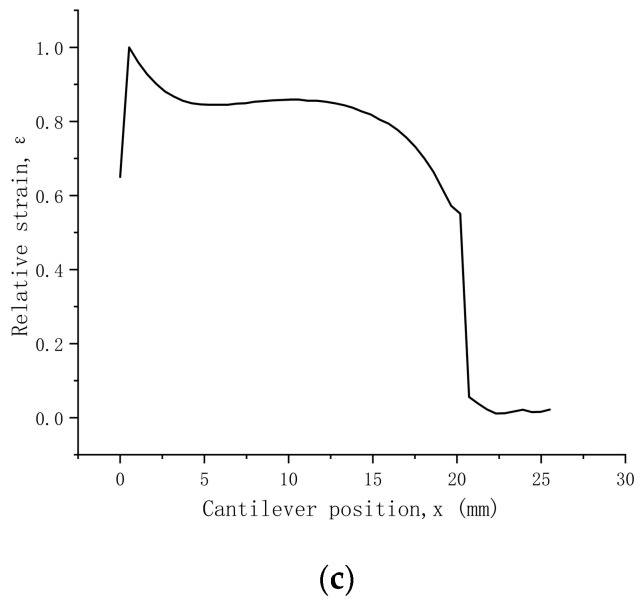
Relative strain along the centerline for differently shaped beams. (**a**) Rectangular structure. (**b**) Trapezoidal structure. (**c**) Triangular structure.

According to the simulation results of the quartz wafers in the Figure 7 and Figure 8, when moving from the free end to the fixed end, the strain increased and peaked at x = 0.5. Only 38% of the quartz wafers in the rectangular structure had a relative strain above 0.6; these values were 53% for the trapezoidal structure and 71% for the triangular structure. Hence, the triangular structure displayed the most favorable average stress distribution among the three shapes. Following the principles of the piezoelectric effect, the electrode ideally should be positioned as close as feasible to the location with the highest strain, potentially enhancing output efficiency. Nevertheless, in real-world applications, controlling the fixed-end width of quartz poses challenges, leading to significant discrepancies in the force–frequency coefficient of rectangular quartz resonators. Therefore, triangular quartz wafers provide a promising research direction for enhancing output efficiency.

### 2.4. Electrode of Quartz Wafers

The cutoff frequency of the electrode region is $f_{s}$, the cutoff frequency of the non-electrode region is $f_{u}$, and the frequency of the elastic wave is f, so there are three different propagation conditions when the elastic wave propagates on the chip, as follows:

(1) When $f<f_{s}<f_{u}$, the elastic wave cannot propagate in the chip.
(2) When $f>f_{u}>f_{s}$, the elastic wave can propagate freely in both the electrode region and the non-electrode region.
(3) When $f_{s}<f<f_{u}$, the elastic wave can only propagate freely in the electrode region and can hardly propagate in the non-electrode region, and the resonant frequency of the elastic wave is in this range. Most of the vibration energy is trapped in the electrode region, and only a tiny part of the energy passes through the boundary into the non-electrode region. The energy entering the non-electrode region will decay exponentially according to the distance away from the electrode region boundary.

**Figure 9:**
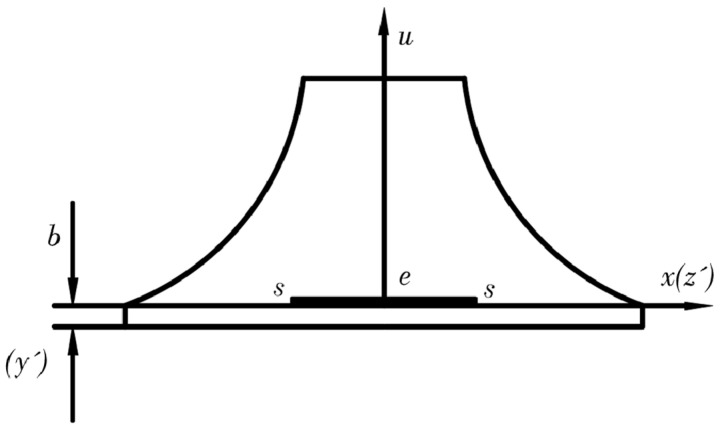
Schematic diagram of resonant wave propagation of a circular quartz resonator.

When the electric field is applied to the thickness direction of the quartz wafer, the thickness-shear vibration, that is, the elastic shear wave, will be stimulated. When electrodes are attached to the wafer surface, the presence of electrodes causes a local mass load, which changes the elastic properties slightly. This change causes the elastic wave to travel somewhat slower in the electrode region, and, therefore, the cutoff frequency in the region is correspondingly reduced. Correspondingly, the cutoff frequency in the non-electrode region is higher. This phenomenon leads to a particular situation: when the frequency of the wave is between the cutoff frequency of the electrode region and the cutoff frequency of the non-electrode region, the wave can only propagate freely in the electrode region, but it can hardly propagate in the non-electrode region. Therefore, the vibration in the electrode region forms a standing wave, while the non-electrode region is exponentially decayed, as shown in Figure 9. This phenomenon is known as the energy trapping effect, which means that the energy of the elastic wave is mainly concentrated in the electrode region, and the wave’s energy decreases rapidly after leaving the electrode region [24].

The boundary conditions of the quartz chip were set in the COMSOL Multiphysics 6.0. In solid mechanics, a fixed constraint is set at one end, and a load of the same magnitude is applied to different locations at the other end. The exact boundary load was set in three places of 5.5 mm, 6 mm, and 6.5 mm at the fixed end of the quartz wafer. The boundary load size was 0.01 N, and 25 MHz was selected as the characteristic frequency for analysis to find the appropriate characteristic frequency. This operation laid the foundation for subsequent analysis and contributed to a more complete understanding of the force–frequency characteristics of cantilevered quartz wafers.

The output energy of quartz is mainly concentrated at the electrode, and this energy localization effect is primarily determined by the propagation characteristics of shear waves in the quartz crystal, that is, the energy trapping theory. According to this theory, when the elastic wave propagates and forms a resonance in the wafer, the wave can travel freely and form a standing wave in the electrode region. At the same time, it decays exponentially in the non-electrode region. This causes the vibration energy to be concentrated in the electrode’s central area and decrease rapidly as it moves away from the central region.

**Figure 10:**
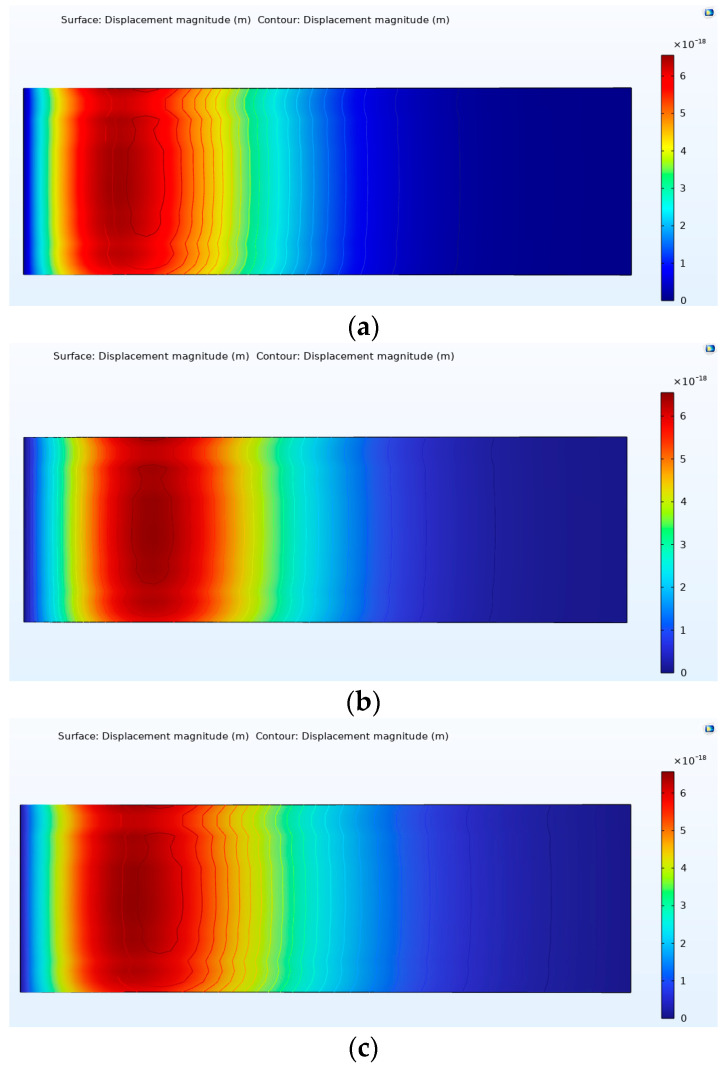
Analysis results of quartz wafers under different boundary loads. (**a**) 6.5 mm. (**b**) 6.0 mm. (**c**) 5.5 mm.

Figure 10 show the finite element analysis results. The results show that, as the applied force gradually approaches the fixed end, the maximum energy of the resonator moves toward the free end. Therefore, when designing the electrode of the cantilever quartz chip, the electrode position can be transferred to the center of the chip near the fixed end.

The finite element analysis results are shown in Table 2. When the same load is applied to the quartz wafer at different force application positions, and when the free end of the force application position moves to the fixed end, the maximum vibration energy position of the quartz wafer moves from 1.26 mm away from the fixed end to 0.15 mm, each time the force application position moves to the fixed end. The stress at the place of maximum vibration energy decreases as the position of force application moves to the fixed end. When the position of force application is at the free end, the stress of the quartz chip is the largest.

The force–frequency coefficient increases as the quartz wafer’s electrode moves closer to the fixed end, indicating improved force–frequency characteristics. To confirm the correlation between the force–frequency coefficient and the quartz’s stress distribution, experiments were conducted to investigate the relationship between the quartz electrode position and the force–frequency coefficient under a rectangular structure.

**Figure 11:**
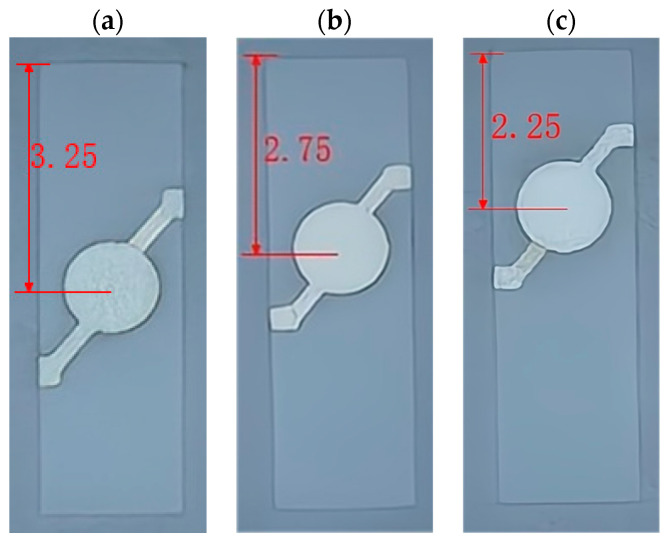
Quartz wafer with different electrode positions: (**a**) 3.25 mm, (**b**) 2.75 mm, and (**c**) 2.25 mm.

Experimental tests were conducted on quartz wafers measuring 6.5 mm × 2 mm and operating at frequencies of 25 MHz to validate further the results obtained from previous simulation analysis. First, the electrodes were in a circular pattern with a diameter of 1.3 mm and were placed 3.25 mm, 2.75 mm, and 2.25 mm from one side of the quartz crystal, as shown in Figure 11. Subsequently, these quartz wafers were placed on cantilever beams at fixed positions ranging from 0.3 mm to 1.1 mm for testing. The force–frequency coefficients of these wafers were then measured to evaluate their response under various conditions, including sensitivity to external pressure and stability under different fixed positions. These experimental findings will aid in determining the optimal electrode mounting position and cantilever conditions required for optimizing sensor performance and accuracy.

## 3. Results

### 3.1. Force–Frequency Coefficient Formula Verification

Table 3 shows that the theoretical values calculated by the force–frequency coefficient formula after coefficient correction were 92% similar to the measured values. Therefore, the force–frequency coefficient calculation formula with coefficient correction could be applied to subsequent calculations for cantilever quartz wafers.

### 3.2. Effect of Shape on the Force–Frequency Coefficient of the Quartz Wafer

**Figure 12:**
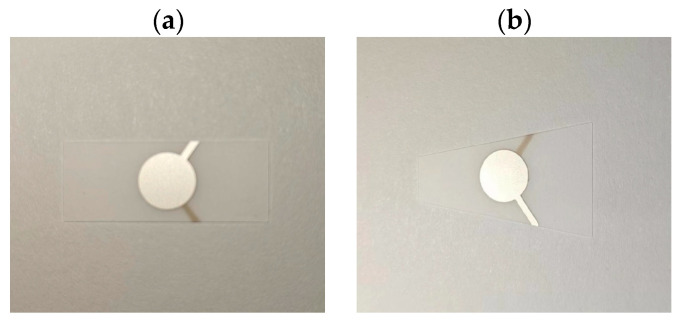
Different shapes of quartz wafers: (**a**) rectangular wafer; (**b**) trapezium wafer.

The rectangular and trapezoidal shapes of the quartz wafer are analyzed experimentally. One end of the trapezoidal and trapezoidal shapes of the quartz wafer is fixed, and the load is applied to one end. Figure 12 shows a physical diagram of two different shapes of quartz wafers.

The load is applied to two kinds of quartz wafers at different positions, and the force–frequency coefficients of two types of quartz wafers with other shapes are obtained according to the recorded data. According to Equation (22), the force applied to the free end results in a force–frequency coefficient of 2690 Hz/N for the quartz chip. However, according to the data in Table 4, when the position of the applied force is at the free end, the force–frequency coefficient of the quartz wafer is 2918 Hz/N. The coincidence between the two values reached 92.18%. Therefore, the experimental results agree with the calculated values, indicating that the force–frequency coefficient of the quartz chip is within the expected range. The experimental data are shown in the Table 4. The experimental results show that the force–frequency coefficient of the rectangular wafer applied at 1.0 mm is 6.4% smaller than that applied at 0.5 mm, and the force–frequency coefficient of the rectangular wafer applied at 1.5 mm is 12.0% smaller than that applied at 1.0 mm. The force–frequency coefficient of the trapezoidal wafer applied load at 1.0 mm is 6.3% smaller than the applied load at 0.5 mm, and the force–frequency coefficient of the load applied at 1.5 mm is 9.9% smaller than the applied load at 1.0 mm. The force position affects the force–frequency coefficient of the trapezoidal chip less than that of the rectangular chip. Therefore, in the subsequent sensor design, the range of the force–frequency coefficient can be stabilized by changing the position of the force application.

### 3.3. Effect of Quartz Wafer Electrode Position on Force–Frequency Coefficient

When the length of the fixed end of the quartz wafer is constant, the force–frequency coefficient of the quartz wafer with the electrode position near the fixed end of 1.0 mm is 5% higher on average than that of the quartz wafer with the electrode position near 0.5 mm. The force–frequency coefficient is 10% higher on average than that of the quartz wafer with the electrode position in the center of the quartz wafer. When the electrode position of the quartz is determined, the force–frequency coefficient of the quartz wafer has a linear relationship with the length of the fixed end, and the force–frequency coefficient decreases with the increase in the size of the fixed end.

**Figure 13:**
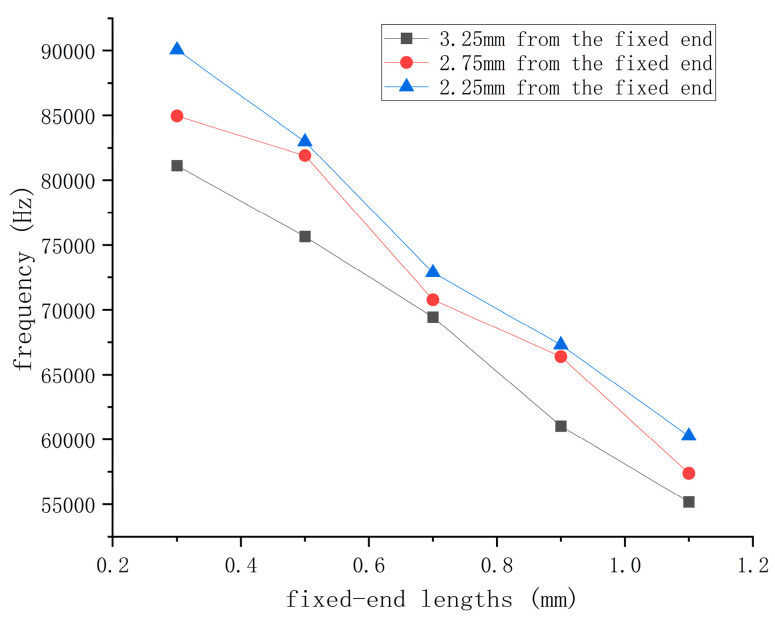
Force–frequency coefficients of quartz wafers with different fixed-end lengths.

Figure 13 shows that, when the fixed-end length structure is the same, the force–frequency coefficient of the quartz wafer increases, the closer the electrode is to the fixed end. When the quartz electrode position is fixed, the force–frequency coefficient of the quartz decreases as the length of the fixed end increases. This finding aligns with the earlier simulation results for rectangular quartz wafers, where a higher relative stress value correlated with a more significant force–frequency coefficient.

## 4. Conclusions

This paper investigated the formula for computing the force–frequency coefficient of cantilever quartz wafers and conducted experiments to validate the revised formula. A formula for calculating the force–frequency coefficient of cantilever quartz resonators was derived, building upon the formula for determining the force–frequency coefficient of quartz resonators with fixed ends. The experimental results showed that the agreement between the calculated values and the actual measured data was more than 92%. The stress distribution of quartz wafers with different shapes under a cantilever beam structure was analyzed using simulations. They also showed that the stress distribution of trapezoidal quartz wafers was more suitable for cantilever beam structures than rectangular quartz wafers.

Furthermore, experimental observations confirmed that the quartz wafer’s force–frequency coefficient increased as the electrode approached the fixed end, while extending the length of the fixed end decreased the force–frequency coefficient. The results showed that, as the applied force gradually approached the fixed end, the maximum energy of the resonator moved to the free end. However, the central position was still concentrated in the center of the quartz chip near the fixed end. Therefore, when designing the electrode of the cantilever quartz chip, the electrode position can be considered to move to the fixed end.

## Figures and Tables

**Table 1:** Dimensions of the fixed and free ends under different cantilever beam structures.

Structure	$b_{fixed}$ (mm)	$b_{free}$ (mm)
Rectangle	10	10
Trapezoid	15	5
Triangle	15	0

**Table 2:** The position of maximum vibration energy of quartz wafer under different applied force positions.

Application Position/mm	5.5	6.0	6.5
The maximum vibration energy position is the distance from the fixed end/mm	1.55	1.41	1.26
The magnitude of stress at the maximum vibration energy/(×10^−3^ N/m^2^)	15.0347	15.0385	15.0405

**Table 3:** Force–frequency coefficients under different sizes.

	1	2	3	4	5
Cantilever beam length (mm)	6.2	6.0	5.8	5.6	5.4
Actual force–frequency coefficient (Hz/N)	107,458	102,007	95,788	87,383	81,521
Theoretical force–frequency coefficient (Hz/N)	100,183	96,952	93,720	90,488	87,256
Accuracy	92.74%	94.79%	97.80%	96.57%	93.43%

**Table 4:** The force–frequency coefficients of two shapes of quartz wafers under different force application positions.

Application Position/mm	0.5	1.0	1.5
rectangle	2750	2574	2265
trapezium	2694	2523	2272

## Data Availability

The original contributions presented in the study are included in the article; further inquiries can be directed to the corresponding author.
